# Persistent intrathecal interleukin-8 production in a patient with SARS-CoV-2-related encephalopathy presenting aphasia: a case report.

**DOI:** 10.1186/s12883-021-02459-3

**Published:** 2021-11-02

**Authors:** Takuya Kudo, Yuichi Hayashi, Kenjiro Kunieda, Nobuaki Yoshikura, Akio Kimura, Mika Otsuki, Takayoshi Shimohata

**Affiliations:** 1 Department of Neurology, Gifu University Graduate School of Medicine, 1-1 Yanagido, 501-1194 Gifu, Japan; 2 Faculty of Health Sciences, Graduate School of Sciences, Hokkaido University, Kita 15, Nishi 7, Kitaku, 060-8638 Sapporo, Japan

**Keywords:** SARS-CoV-2, Encephalopathy, IL-8, Aphasia, Case report

## Abstract

**Background:**

Neurological manifestations of coronavirus disease 2019 (COVID-19) are increasingly recognized and include encephalopathy, although direct infection of the brain by SARS-CoV-2 remains controversial. We herein report the clinical course and cytokine profiles of a patient with severe SARS-CoV-2-related encephalopathy presenting aphasia.

**Case presentation:**

An 81-year-old man developed acute consciousness disturbance and status epileptics several days after SARS-CoV-2 infection. Following treatment with remdesivir and dexamethasone, his consciousness and epileptic seizures improved; however, amnestic aphasia and agraphia remained. Two months after methylprednisolone pulse and intravenous immunoglobulin, his neurological deficits improved. We found increased levels of interleukin (IL)-6, IL-8, and monocyte chemoattractant protein-1 (MCP-1), but not IL-2 and IL-10 in the serum and cerebrospinal fluid (CSF), and the levels of serum IL-6 and MCP-1 were much higher than those in the CSF. The level of IL-8 in the CSF after immunotherapy was four times higher than that before immunotherapy.

**Conclusion:**

The cytokine profile of our patient was similar to that seen in severe SARS-CoV-2-related encephalopathy. We demonstrated (i) that the characteristic aphasia can occur as a focal neurological deficit associated with SARS-CoV-2-related encephalopathy, and (ii) that IL8-mediated central nervous system inflammation follows systemic inflammation in SARS-CoV-2-related encephalopathy and can persist and worsen even after immunotherapy. Monitoring IL-8 in CSF, and long-term corticosteroids may be required for treating SARS-CoV-2-related encephalopathy.

**Supplementary Information:**

The online version contains supplementary material available at 10.1186/s12883-021-02459-3.

## Background

Neurological manifestations of coronavirus disease 2019 (COVID-19) are increasingly recognized and include encephalopathy, although direct infection of the brain by SARS-CoV-2 remains controversial [1]. Aphasia has been reported as a symptom of SARS-CoV-2-related encephalopathy [2, 3] or SARS-CoV-2-related ischemic stroke [4, 5]. Therefore, discrimination between encephalopathy and stroke is very important to treat aphasia. We herein report the clinical course and cytokine profiles of a patient with severe SARS-CoV-2-related encephalopathy presenting aphasia.

## Case presentation

An 81-year-old man was hospitalized because of a SARS-CoV-2 infection confirmed by real time-polymerase chain reaction (RT-PCR) using a nasopharyngeal swab sample. His fever and respiratory symptoms improved within 10 days following remdesivir and dexamethasone treatment (6 mg/day, 10 days). Eight days after the diagnosis, he developed acute consciousness disturbance, status epileptics, and urinary incontinence.

**Fig. 1:**
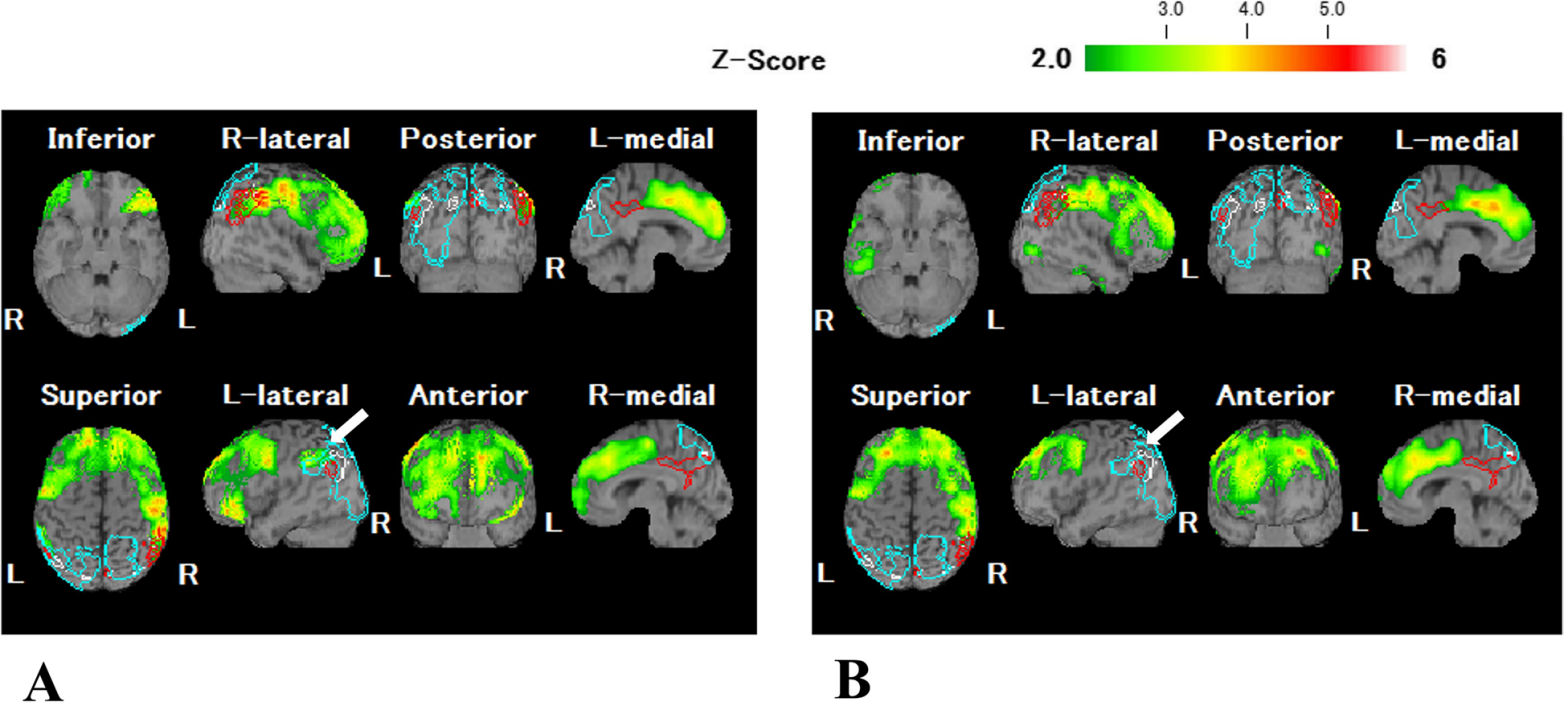
Serial single photon emission computed tomography (SEPCT) assessment. The easy Z-score analysis images for SPECT obtained 1 month (**A**) and 2 months (**B**) after the neurological symptom onset. Panel **A** shows decreased regional cerebral blood flow (rCBF) in the left supramarginal gyrus **(arrow**) in addition to the bilateral frontal cortices. Panel **B** shows improved rCBF in the left supramarginal gyrus (**arrow**)

He was healthy man without neurological complication including dementia, seizure, or urinary incontinences before SARS-CoV-2 infection. Brain MRI showed bilateral mild frontal and temporal cortical atrophies with moderate deep white matter hyperintense lesions. These white matter lesions were not detected on diffusion-weighted MRI. Routine CSF analysis including cell counts and total protein, were normal. Oligoclonal band (OCB) was negative. IgG index and CSF/serum albumin ratio (Q_alb_) was 0.43 and 4.3 × 10^− 3^, respectively. These parameters were not elevated. RT-PCR for SARS-CoV-2 was not performed by CSF. We diagnosed him with SARS-CoV-2-related “possible autoimmune encephalitis” based on the criteria [6]. Subsequently, we initiated three courses of intravenous methylprednisolone pulse (IVMP; 1000 mg/day, 3 days) and intravenous immunoglobulin therapy (0.4 g/kg/day, 5 days). Corticosteroids were gradually tapered from 1 mg/kg/day. Following the treatments, his consciousness level markedly improved, and epileptic seizures were well controlled; however, amnestic aphasia and agraphia [particularly predominant in Kana (phonogram)] remained. His aphasia was improved 2 months after immunotherapy. The second CSF analysis showed normal cell counts (3 /μL) and total protein (21 mg/dL). Although no obvious changes were noted on serial MRI assessments, including diffusion-weighted images, single-photon emission computed tomography (SPECT) performed 1 month after the onset of neurological symptoms revealed decreased regional cerebral blood flow (rCBF) in the left supramarginal gyrus (**arrow**) in addition to the bilateral frontal cortices (Fig. 1**, panel A)**. SPECT study performed 2 months after the neurological symptom onset, revealed improved rCBF in the left supramarginal gyrus, reflecting improvement in his aphagia (Fig. 1**,** panel B).

To distinguish encephalopathy from infectious encephalitis [7], we analyzed the levels of interleukin (IL)-2, IL-6, IL-8, IL-10, and monocyte chemoattractant protein-1 (MCP-1) by enzyme-linked immuno-sorbent assay, enzyme immunoassay, or chemiluminescent enzyme immunoassay in the serum and CSF before and 3 weeks after immunotherapy (Supplemental Table 1). Supplemental Table 1 shows the results of the cytokine analyses. Increased levels of IL-8, and MCP-1 but not IL-2 and IL-10 were observed in both the serum and CSF. IL-6 levels were increased only in the serum. The levels of serum IL-6 and MCP-1 were much higher than those in the CSF. IL-8 levels in the CSF after immunotherapy (336 pg/mL) were four times higher than those prior treatment (83.6 pg/mL), which in turn were higher than concentrations observed in the serum (18 pg/mL before, and 28.7 pg/mL 3 weeks after immunotherapy). Therefore, corticosteroids were continued, and the patient remained under close observation.

## Discussion and conclusion

We report a patient with SARS-CoV-2 infection presenting characteristic aphasia and identify that the pathophysiology was similar to encephalopathy associated with systemic inflammation based on the results of the cytokine analysis showing higher serum, than CSF levels of IL-6 and MCP-1. The cytokine profile of our patient was similar to that seen in severe SARS-CoV-2-related encephalopathy [7].

We demonstrated that two novel findings regarding SARS-CoV-2-related encephalopathy. First, we showed that characteristic aphasia could occur as a focal neurological deficit associated with SARS-CoV-2-related encephalopathy using SPECT. Although aphasia associated with SARS-CoV-2-related encephalopathy [2, 3] or secondary ischemic stroke has been reported [4, 5], focal lesions on imaging have not been demonstrated (Supplemental Fig 1). Additionally, MR angiography showed no stenosis or occlusion (Supplemental Fig. 1). However, in our patient, both the aphasia and SPECT findings improved with treatment, suggesting that focal lesions can occur with encephalopathy, although the pathogenesis is unknown.

Second, we demonstrated that IL8-mediated central nervous system (CNS) inflammation follows systemic inflammation in SARS-CoV-2-related encephalopathy and might persist and worsen even after immunotherapy. Additionally, we considered that the chronic intrathecal production of IL-8 might be associated with longer neurological complication in his clinical course.

IL-8 is a chemoattractant of neutrophils with the ability to eliminate cells infected with virus or bacteria. The intrathecal IL-8 production is various diseases or conditions in the CNS including SARS-CoV-2-related stroke [8]. In the current patient, stroke and infectious meningitis were excluded by his MRI findings and CSF data. As his data was normal cell counts, unelevated IgG index and negative result of OCB, we considered that his condition was encephalopathy rather than encephalitis. However, Q_alb_ was not elevated in his samples. The intrathecal production of IL-8 is usually due to microglial activation [9], which has been reported in post-mortem studies of SARS-CoV-2-related encephalopathy [1].

Thus, systemic inflammation may lead to persistent activation of microglia. Recently, transcriptome analyses showed broad cellular perturbations indicating that barrier cells of the choroid plexus sense and relay peripheral inflammation into the brain, was observed in patients with severe SARS-CoV-2-related encephalopathy [10].

These findings are interesting because it has recently been pointed out that SARS-CoV-2 infection can cause microglial activation, which may have long-term effects on immune processes in the CNS, resulting in brain fog and cognitive impairment [11]. Although we did not confirm further changes in IL-8 over time in our patient, careful monitoring of CSF IL-8 and long-term steroids should be considered in SARS-CoV-2-related encephalopathy.

## Supplementary Information

**Additional file 1: Supplementary Table 1**. Cytokine analysis data

**Additional file 2: Supplemental Figure 1.** MR images including MR angiograph. A: Diffusion-weighted images; B: FLAIR images; C: MRA. R means the right side.

## Data Availability

Further clinical data are available from the corresponding author upon reasonable request.

## Abbreviations

COVID-19: Coronavirus disease 2019
CNS: Central nervous system
CSF: Cerebrospinal fluid
IL: Interleukin
IVMP: Intravenous methylprednisolone pulse
MCP-1: Monocyte chemoattractant protein-1
OCB: Oligoclonal band
Q_alb_: CSF/serum albumin ratio
rCBF: Regional cerebral blood flow
RT-PCR: Real time-polymerase chain reaction
SPECT: Single-photon emission computed tomography
