# Supplementary figures and images for: Persistent intrathecal interleukin-8 production in a patient with SARS-CoV-2-related encephalopathy presenting aphasia: a case report
Source: BMC Neurol. 2021 Nov 2;21:426. doi: 10.1186/s12883-021-02459-3 (PMC8560881; doi:10.1186/s12883-021-02459-3)

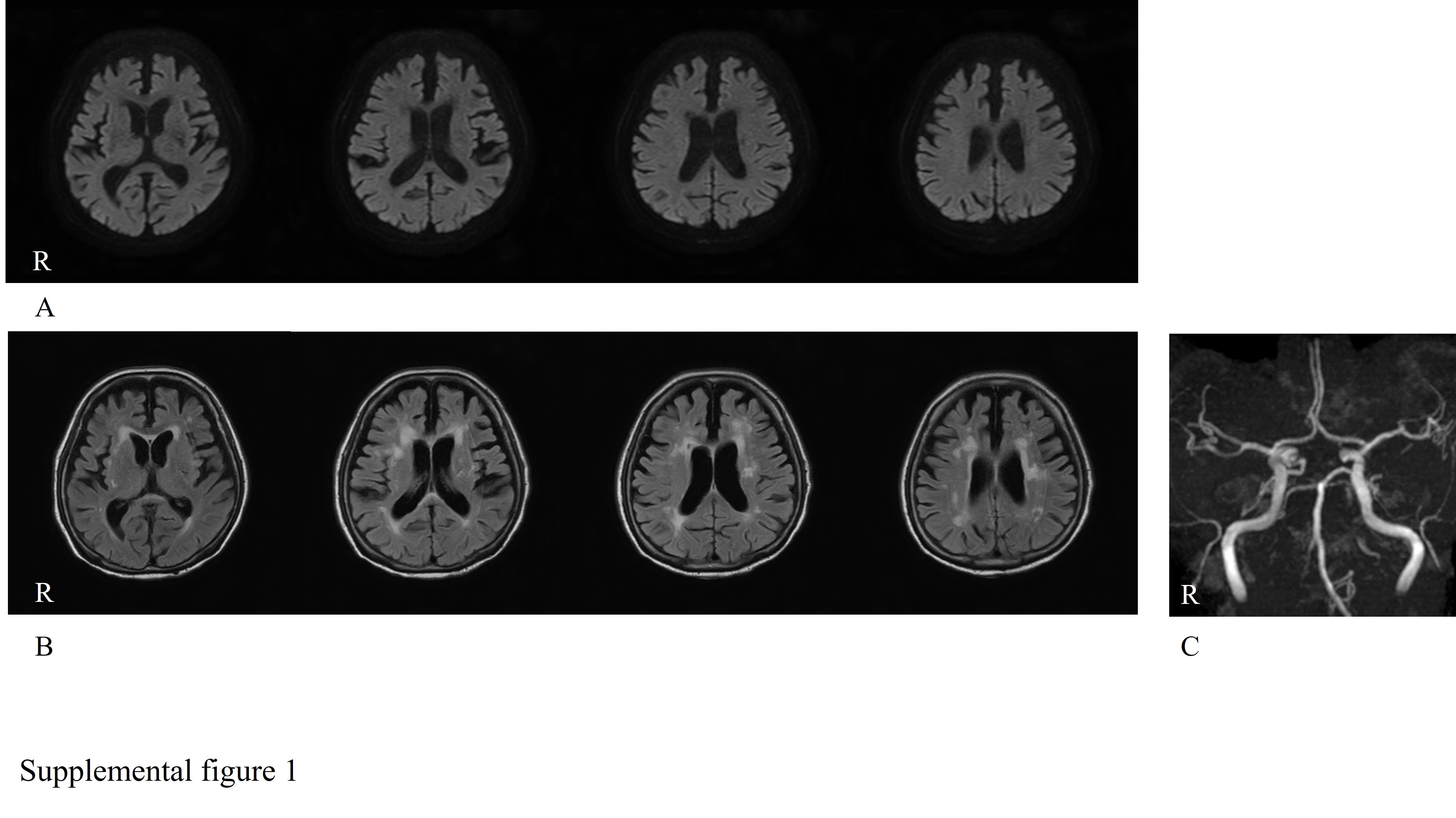

Supplement: Supplementary file 2 — Additional file 2: Supplemental Figure 1. MR images including MR angiograph. A: Diffusion-weighted images; B: FLAIR images; C: MRA. R means the right side. [file 12883_2021_2459_MOESM2_ESM.jpg]
